# Reviewers BJCVS 32.4

**Published:** 2017

The Brazilian Journal of Cardiovascular Surgery (BJCVS) is grateful for the reviewers, listed below, which collaborated in this edition. Without their work would be impossible to keep the high scientific standard of our journal.

Aubyn Marath
Beatriz Furlanetto
Bruno Rocha
Carla Tanamati
Fabio Navarro
Francisco Costa
Gabriel Liguori
Luciano Albuquerque
Magaly Arrais dos Santos
Marcos Aurelio Barboza de Oliveira
Nelson Hossne
Noedir Stolf
Omar Mejía
Paulo Roberto Evora
Rachel Nina
Renato Arnoni
Ricardo Benfatti
Ricardo Lima
Rodrigo Jaenisch
Victor Ferreira

